# Crystal structure of *fac*-aqua­[(*E*)-4-(benzo[*d*]thia­zol-2-yl)-*N*-(pyridin-2-yl­methyl­idene)aniline-κ^2^ *N*,*N*′]tricarbonylrhenium(I) hexa­fluorido­phosphate methanol monosolvate

**DOI:** 10.1107/S2056989019004298

**Published:** 2019-04-05

**Authors:** Ioanna Roupa, Michael Kaplanis, Catherine Raptopoulou, Maria Pelecanou, Ioannis Pirmettis, Minas Papadopoulos, Vassilis Psycharis

**Affiliations:** a Institute of Nuclear and Radiological Sciences and Technology, Energy and Safety, National Centre for Scientific Research "Demokritos", 15310 Athens, Greece; b Institute of Nanoscience and Nanotechnology, Department of Materials Science, National Centre for Scientific Research "Demokritos", 15310 Athens, Greece; c Institute of Biosciences & Applications, National Centre for Scientific Research "Demokritos", 15310 Athens, Greece

**Keywords:** crystal structure, tricarbonyl rhenium (I), mixed ligand complex, 2-(4′-amino­phen­yl)benzo­thia­zole, *trans* effect, Hirshfeld surface analysis

## Abstract

In the title compound, *fac*-[Re(C_19_H_13_N_3_S)(CO)_3_(H_2_O)]PF_6_·CH_3_OH, the coordination environment of the Re^I^ atom is octa­hedral with a C_3_N_2_O coordination set. In this mol­ecule, the *N*,*N*′ bidentate ligand, (*E*)-4-(benzo[*d*]thia­zol-2-yl)-*N*-(pyridin-2-yl­methyl­idene)aniline, and the monodentate aqua ligand occupy the three available coordination sites of the [Re(CO)_3_]^+^ core, generating a ‘2 + 1’ mixed-ligand complex. In this complex, the Re—C bonds of the carbonyl ligands *trans* to the coordinating *N*,*N*′ atoms of the bidentate ligand are longer than the Re—C bond of the carbonyl group *trans* to the aqua ligand, in accordance with the intensity of their *trans* effects. The complex is positively charged with PF_6_^−^ as the counter-ion. In the structure, the complexes form dimers through π–π inter­molecular inter­actions. O—H⋯O and O—H⋯N hydrogen bonds lead to the formation of stacks parallel to the *a* axis, which further extend into layers parallel to (01). Through O—H⋯F hydrogen bonds between the complexes and the PF_6_^−^counter-anions, a three-dimensional network is established.

## Abstract 2

A structural *trans* effect study and the packing arrangement of a *fac-*tricarbonyl Re^I^ ‘2 + 1’ mixed-ligand complex are reported.

## Chemical context   

‘2 + 1’ mixed-ligand complexes of general formula *fac*-[*M*(CO)_3_ *L*_1_ *L*_2_], where *M* is Re or ^99m^Tc, *L*_1_ is a bidentate ligand (bi­pyridine, 2-picolinic acid, acetyl­acetone, *etc*) and *L*_2_ is a monodentate ligand (aqua, imidazole, phosphine or isocyanide), have been studied extensively for the development of novel radiopharmaceuticals for diagnosis (*M* = ^99m^Tc) or radiotherapy (*M* = ^186/188^Re) (Knopf *et al.*, 2017 ▸; Mundwiler *et al.*, 2004 ▸; Papagiannopoulou *et al.*, 2014 ▸; Tri­antis *et al.*, 2013 ▸; Shegani *et al.*, 2017 ▸). Furthermore, recent studies have revealed the potential of such *fac*-[Re(CO)_3_ *L*_1_ *L*_2_] complexes as anti­cancer agents (Leonidova & Gasser, 2014 ▸). According to the ‘2 + 1’ strategy, the inter­mediate aqua complex *fac*-[Re(CO)_3_(*L*_2_)(H_2_O)] plays a crucial role. The labile water ligand can readily be substituted by a monodentate ligand *L*_2_ (typically heterocyclic aromatic amines, isocyanides, phosphines), generating the final *fac*-[Re(CO)_3_ *L*_2_ *L*_1_] product in high yield. The ‘2 + 1’ complexes are characterized by kinetic stability and structural variability that facilitates the tuning of physicochemical properties and tethering of pharmacophores of inter­est towards the generation of targeted multifunctional compounds.
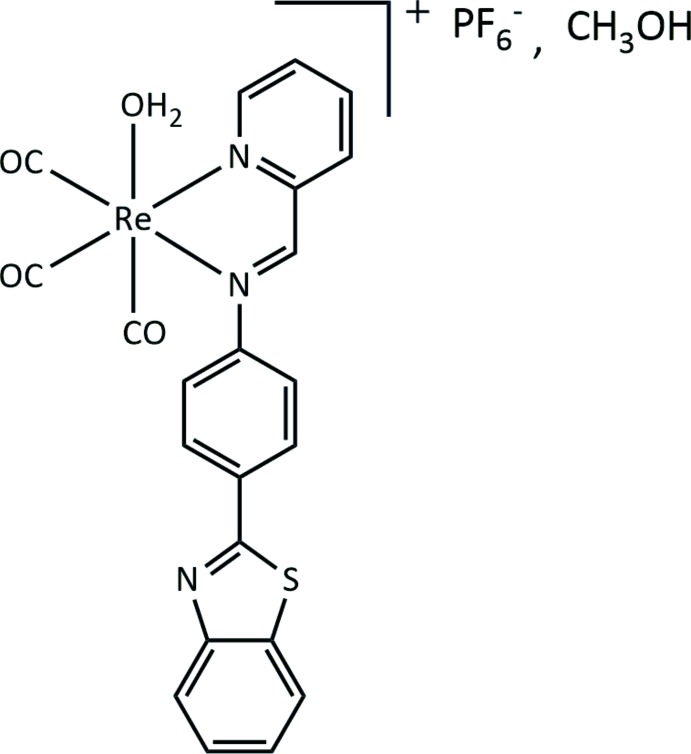


As part of our ongoing research in the field of Re/Tc coordination chemistry, we report herein the structure of the ‘2 + 1’ tricarbonyl rhenium(I) complex *fac*-[Re(CO)_3_(NNbz)(H_2_O)]PF_6_·CH_3_OH where the bidentate NNbz ligand is (*E*)-4-(benzo[*d*]thia­zol-2-yl)-*N*-(pyridin-2-yl­methyl­idene)aniline. The NNbz ligand carries the 2-(4′-amino­phen­yl)benzo­thia­zole scaffold, which also exhibits inter­esting biol­ogical properties against a variety of targets and presents great potential for diagnostic/therapeutic applications (Keri *et al.*, 2015 ▸; Kiritsis *et al.*, 2017 ▸; Bradshaw & Westwell, 2004 ▸).

## Structural commentary   

**Figure 1:**
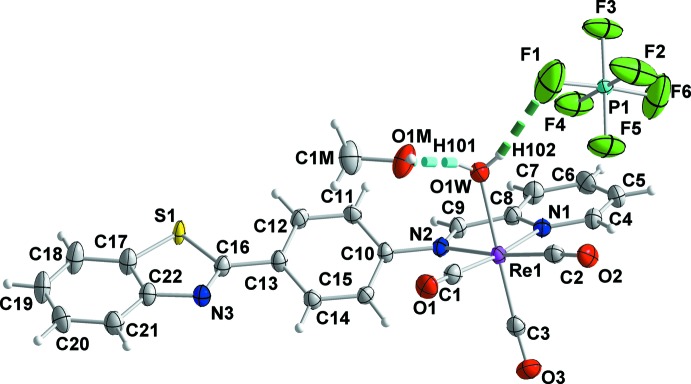
Mol­ecular structure and labeling scheme for the title Re^I^ complex, the methanol solvent mol­ecule and the PF_6_^−^ counter-anion. Displacement ellipsoids are drawn at the 50% probability level. Cyan and dark-green dashed lines indicate the O1*W*—H101⋯O1*M* and O1*W*—H102⋯F1 hydrogen bonds, respectively.

The asymmetric unit of the title compound comprises one *fac*-aqua­tricarbonyl-(*E*)-4-(benzo[*d*]thia­zol-2-yl)-*N*-(pyridin-2-yl­methyl­idene)aniline–rhenium(I) complex mol­ecule, one PF_6_^−^ counter-anion and one methanol solvent mol­ecule (Fig. 1 ▸). Within the complex, the Re^I^ atom presents a distorted octa­hedral C_3_N_2_O coordination set with the three tricarbonyl ligands in facial and the bidentate di­imine (NNbz) and the monodentate water ligands in a *cis* arrangement (Fig. 1 ▸). The two coordinating nitro­gen atoms N1 and N2 of the bidentate NNbz ligand together with two carbonyl carbon atoms define the equatorial plane with almost perfect planarity (deviation from the least-squares plane = 0.006 Å). The Re—N1 and Re—N2 distances are 2.177 (2) and 2.194 (2) Å, respectively. The oxygen atom of the water mol­ecule [Re—O1*W* = 2.189 (2) Å] and the carbon atom from the third carbonyl ligand define the axial direction of the octa­hedron. Both the Re—N and the Re—O distances fall in the range of observed values in complexes with a di­imine, aqua or tricarbonyl core (Mella *et al.*, 2016 ▸; Connick *et al.*, 1999 ▸; Schutte *et al.* 2011 ▸; Salignac *et al.*, 2003 ▸; Knopf *et al.*, 2017 ▸; Rillema *et al.*, 2007 ▸; Barbazán *et al.*, 2009 ▸; Carrington *et al.*, 2016 ▸; Tzeng *et al.*, 2011 ▸; Grewe *et al.*, 2003 ▸). The NNbz ligand deviates from planarity as the dihedral angle between the central phenyl ring and the benzo­thia­zole group is 20.48 (8)°, while the dihedral angle between the phenyl ring and the pyridine ring is 39.13 (8)°.

## Supra­molecular features   

**Figure 2:**
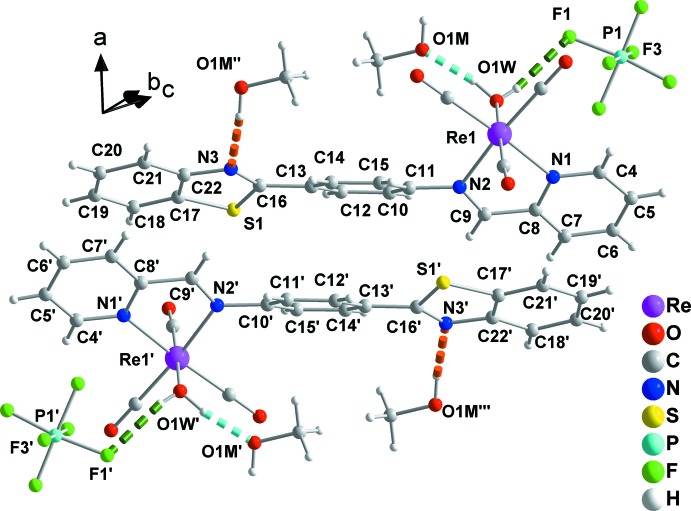
Dimers of complexes formed through π–π overlap between their coordinating NNbz ligands and inter­molecular inter­actions between dimers with methanol solvent mol­ecules and PF_6_^−^ counter-anions. Colour code as in Fig. 1 ▸ with the additional O1*M*—H201⋯N3 inter­actions indicated by orange dashed lines. [Symmetry codes: (′) 1 − *x*, 1 − *y*, 1 − *z*; (′′) 2 − *x*, 1 − *y*, 1 − *z*; (′′′) −1 + *x*, *y*, *z*.]

**Figure 3:**
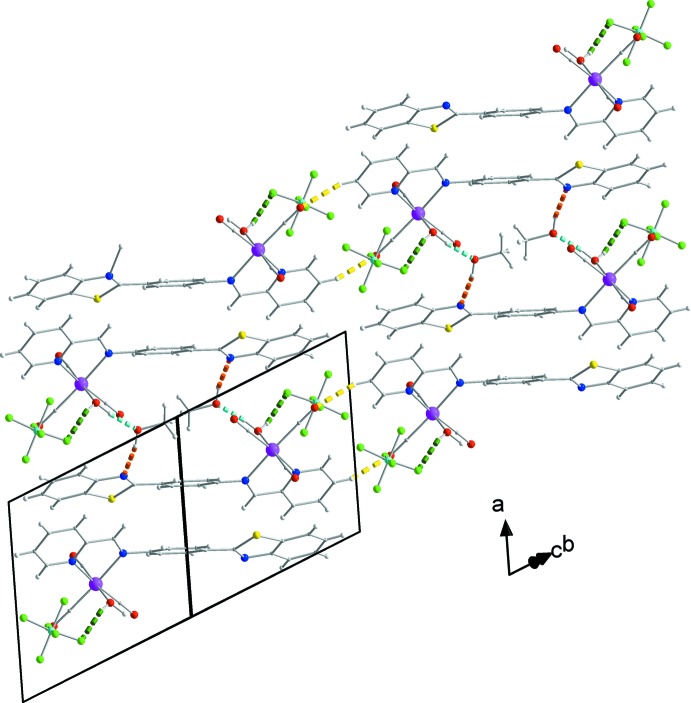
Layers of complexes parallel to (0
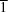
1). C5—H5⋯O2 hydrogen bonds are indicated by yellow dashed lines. For the atoms and the rest of the bonds, the colour code is as in Fig. 2 ▸.

**Figure 4:**
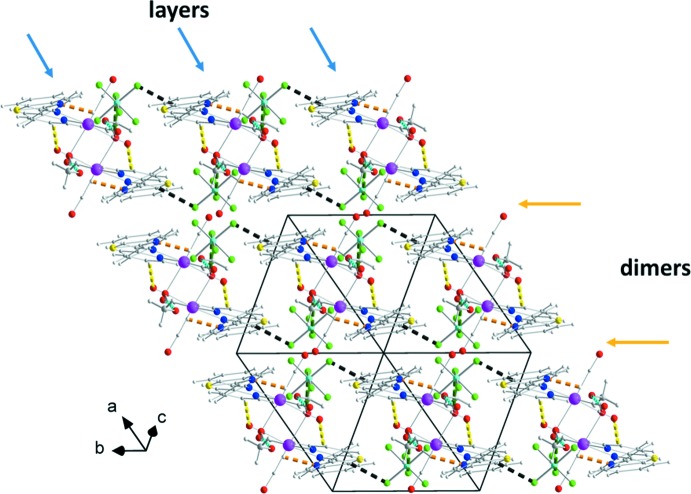
Three-dimensional arrangement of layers. C9—H9⋯F3^ii^ hydrogen bonds are indicated by black dashed lines. For the atoms and the rest of the bonds, the colour code is as in previous figures. The cyan arrows indicate the position of the layers within the structure and the orange ones the areas where the complexes inter­act through π–π inter­actions.

The counter-anion and the methanol solvent mol­ecules form O1*W*—H102⋯F1 and O1*W*—H101⋯O1*M* hydrogen bonds with the aqua ligand (Fig. 1 ▸, Table 1 ▸). Neighbouring complexes present a π–π overlap between their coordinating NNbz ligands, forming dimers (Fig. 2 ▸). More specifically, the mol­ecules are centrosymetrically related and thus exhibit parallel phenyl rings of the NNbz ligand at a distance of 3.50 (1) Å. In addition, both the pyridine rings and the phenyl rings of the benzo­thia­zole parts of neighbouring centrosymmetrically related NNbz ligands overlap with each other, with their respective centroids *Cg*1 and *Cg*2 lying at a distance of 3.8525 (1) Å and forming an angle of 18.67 (6)° [*Cg*1 and *Cg*2′ are the centroids of the N1, C4–C8 and C17′–C22′ rings; symmetry code: (′) 1 − *x*, 1 − *y*, 1 − *z*; Fig. 2 ▸]. The dimers are stacked along the *a-*axis direction. Methanol solvent mol­ecules are inter­leaved between adjacent dimers within the stacked mol­ecules and are linked through inter­molecular O1*W*—H101⋯O1*M* and O1*M*—H201⋯N3 inter­actions (Fig. 3 ▸). These stacks are extended into layers parallel to (0
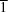
1) through C5—H5⋯O2 hydrogen bonds and further O1*W*—H102⋯F1, C9—H9⋯F3^ii^ (Table 1 ▸) hydrogen bonds between the counter-anions and the coordinating ligands result in the formation of a three-dimensional network structure (Fig. 4 ▸).

## Hirshfeld surface study   

**Figure 5:**
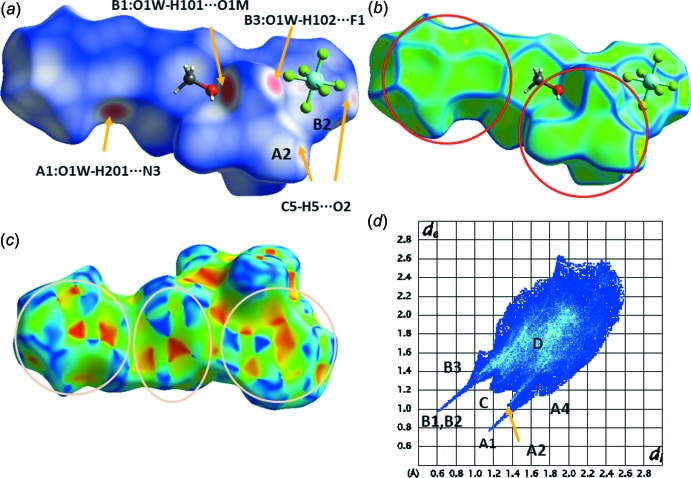
Views of the Hirshfeld surfaces mapped over (*a*) *d*_norm_, (*b*) curvedness and (*c*) shape-index, and (*d*) the fingerprint plot for the title complex. The red circles in (*b*) indicate patches of the same shape corresponding to contact areas of neighbouring complexes. The central ellipse in (*c*) indicates the π–π overlap of the central phenyl rings, and the two circles at both ends of the surface the overlap of the pyridine ring and the phenyl ring of the benzo­thia­zol part of neighbouring centrosymmetrically related NNbz ligands. In (*d*), *d*_e_ and *d*_i_ are the distances to the nearest atom centre exterior and inter­ior to the surface. A1 and A4 stand for the acceptor atoms in O1*W*—H201⋯N3 and C⋯H inter­actions. A2, B2 indicate the acceptor atom and the H-donated atom in the C5—H5⋯O2 inter­action, B1 the H101 atom in the O1*W*—H101⋯O1*M* inter­action, and B3, C and D the H⋯F, H⋯H and C⋯C inter­actions, respectively.

The view of the Hirshfeld surface mapped with *d*_norm_ (Fig. 5 ▸ *a*) reveals almost all of the hydrogen-bonding inter­actions discussed above as intense red areas. The same view of the surface mapped with the curvedness property reveals the contact areas of the tricarbonyl part of the complex with the benzo­thia­zole end of the coordinating ligand, as indicated by patches of the same shape (circled areas in Fig. 5 ▸ *b*). Finally, the plot of the surface mapped with the shape-index property (Fig. 5 ▸ *c*) gives clear evidence that this part of the mol­ecule inter­acts with a centrosymmetrically related neighbour, as the shape of the patterns on the surface are related centrosymmetrically. The rhombic and triangular shapes with the complementary red(hollows)/blue(bumps) colours are characteristic of π–π inter­actions. The asymmetric distribution of points in the fingerprint plot for the complex shown in Fig. 5 ▸ *d* is indicative that there are contributions from different mol­ecules. The relative contributions for the H⋯H, O⋯H, H⋯F, C⋯H and C⋯C inter­actions are 23.2, 20.2, 16.2, 9.7 and 8.2%, respectively, which, in total, amount to 96.4%. The rest of the inter­molecular inter­actions include O⋯S (3.1%), H⋯N (2.3%), C⋯S (2.4%) and C⋯N (1.5%), as well as other inter­actions with <1% contribution.

## Database survey   

A search of the Cambridge Structural Database (Version 5.39, update of August 2918; Groom *et al.*, 2016 ▸) revealed twelve *fac*-aqua­tricarbonyl Re^I^ complexes with different *N*,*N*′-bidentate ligands. A thirteenth structure, FIWQUX-2 (Schutte *et al.*, 2011 ▸), consists of two symmetry-independent complexes. The Re—N bond lengths observed in the present study (Table 2 ▸) are longer than those in most of the previously studied complexes, and close to the longer ones observed in the SEHGUK structure (Knopf *et al.*, 2017 ▸) with the 4,7-diphenyl-1,10-phenanthroline bidentate ligand. As can be seen in Table 2 ▸, the Re—N bond lengths fall in the range 2.142–2.210 Å. The corresponding range for the Re—O1*W* bond is 2.143–2.214 Å, with the value observed in the present study falling in the middle of this range. The values of the Re—C bond lengths are also given. In all cases, the Re—C bonds *trans* to water mol­ecule are shorter than the Re—C bonds *trans* to N atoms, in accordance with the intensity of the *trans* effect of the coordinating ligands.

## Synthesis and crystallization   

A mixture of Re(CO)_5_Br (81 mg, 0.2 mmol) and the NNbz ligand (69 mg, 0.22 mmol) was suspended in 7 ml toluene and refluxed under an N_2_ atmosphere for 4 h. The red suspension was then allowed to cool to room temperature. The red solid that formed was dissolved in aceto­nitrile (25 ml) and a batch of AgPF_6_ (55 mg, 0.22 mmol) was added. The reaction mixture was refluxed for 18 h under an N_2_ atmosphere. The round flask was covered with aluminium foil to avoid exposure to any ambient light. The reaction mixture was allowed to cool for 1 h to 273 K, and then the precipitate (AgBr) was filtered off through celite. The yellow–orange filtrate was evaporated to dryness under reduced pressure, and the residue was recrystallized from aceto­nitrile/water to obtain 67 mg (45% yield) of the aqua complex. Analysis calculated (%) for C_22_H_15_F_6_N_3_O_4_PReS: C, 35.30; H, 2.02; N, 5.61; found: C: 35.43, H: 2.05, N: 5.52. IR (cm^−1^): 2034, 1941, 1914 cm^−1^ (vibration tension of the C≡O bond), 832, 556 cm^−1^ (due to the counter-ion PF_6_^−^). ^1^H NMR (DMSO-*d*_6_), *δ* (ppm): 9.58, 9.15, 8.49, 8.45, 8.37, 8.21, 8.12, 7.98, 7.83, 7.78, 7.60, 7.52. Red–brown crystals suitable for X-ray analysis were obtained by slow evaporation from a methanol/water solution.

## Refinement   

Crystal data, data collection and structure refinement details are summarized in Table 3 ▸. All H atoms were freely refined.

## Supplementary Material

Crystal structure: contains datablock(s) I. DOI: 10.1107/S2056989019004298/wm5494sup1.cif

Structure factors: contains datablock(s) I. DOI: 10.1107/S2056989019004298/wm5494Isup2.hkl

CCDC reference: 1906503

Additional supporting information:  crystallographic information; 3D view; checkCIF report

## Figures and Tables

**Table 1:** Hydrogen-bond geometry (Å, °)

*D*—H⋯*A*	*D*—H	H⋯*A*	*D*⋯*A*	*D*—H⋯*A*
C5—H5⋯O2^i^	0.91 (4)	2.59 (4)	3.439 (4)	156 (3)
C9—H9⋯F3^ii^	0.94 (3)	2.47 (3)	3.390 (3)	166 (2)
O1*W*—H101⋯O1*M*	0.91 (4)	1.67 (4)	2.558 (3)	165 (4)
O1*W*—H102⋯F1	0.72 (4)	2.36 (4)	3.059 (5)	164 (4)
O1*M*—H201⋯N3^iii^	0.88 (5)	2.01 (5)	2.842 (3)	158 (4)

Symmetry codes: (i) 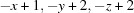; (ii) 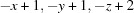; (iii) 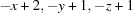.

**Table 2:** Characteristic bond lengths (Å) for a series of Re^I^ complexes with a *fac*-aqua tricarbonyl di­imine octa­hedral core

	Re—N1	Re—C1	Re—N2	Re—C2	Re—O1*W*	Re—C3
Present work	2.177 (2)	1.925 (3)	2.194 (2)	1.920 (3)	2.189 (2)	1.899 (3)
ENAJAG^*a*^	2.156 (7)	1.935 (11)	2.165 (7)	1.884 (10)	2.176 (7)	1.886 (11)
ENAJEK^*a*^	2.173 (5)	1.911 (7)	2.178 (5)	1.921 (7)	2.191 (5)	1.879 (7)
FIWQUX-1^*b*^	2.168 (7)	1.91 (1)	2.180 (5)	1.914 (8)	2.215 (6)	1.88 (1)
FIWQUX-2^*b*^	2.164 (7)	1.902 (10)	2.178 (7)	1.909 (10)	2.210 (6)	1.868 (10)
KAWLO*L* ^*c*^	2.168 (4)	1.914 (6)	2.175 (4)	1.929 (7)	2.162 (3)	1.893 (5)
UHUNOA^*d*^	2.161 (5)	1.938 (7)	2.183 (5)	1.931 (7)	2.181 (5)	1.898 (7)
	2.160 (5)	1.928 (6)	2.174 (4)	1.926 (9)	2.196 (6)	1.915 (7)
SEHGUK^*e*^	2.210 (3)	1.928 (4)	2.200 (3)	1.929 (4)	2.196 (2)	1.896 (4)
PIDYILf^*f*^	2.167 (2)	1.918 (3)	2.167 (2)	1.918 (3)	2.143 (3)	1.912 (4)
UHUNUG^*d*^	2.161 (6)	1.901 (9)	2.165 (6)	1.914 (10)	2.190 (5)	1.882 (10)
	2.165 (6)	1.901 (9)	2.161 (6)	1.91 (1)	2.190 (5)	1.88 (1)
VUDWAT^*g*^	2.185 (4)	1.888 (7)	2.175 (6)	1.925 (8)	2.165 (5)	1.853 (9)
ETEDEO^*h*^	2.186 (5)	1.933 (6)	2.178 (5)	1.902 (7)	2.155 (5)	1.896 (7)
IZORI*Z* ^*i*^	2.203 (3)	1.912 (4)	2.142 (3)	1.922 (4)	2.173 (3)	1.904 (4)
TUTDAN^*j*^	2.168 (6)	1.925 (8)	2.175 (6)	1.913 (9)	2.175 (6)	1.89 (1)

Notes: (*a*) 1,10-Phenanthroline (Connick *et al.*, 1999 ▸); (*b*) 1,10-phenanthroline (Schutte *et al.*, 2011 ▸); (*c*) 1,10-phenanthroline (Schutte *et al.*, 2011 ▸); (*d*) 1,10-phenanthroline (Salignac *et al.*, 2003 ▸); (*e*) 4,7-diphenyl-1,10-phenanthroline (Knopf *et al.*, 2017 ▸); (*f*) 2,2′-bi­pyrazine (Rillema *et al.*, 2007 ▸); (*g*) 2-hy­droxy­benzoic acid hydrazide, (Barbazán *et al.*, 2009 ▸); (*h*) 2-(2′-pyrid­yl)benzo­thia­zole (Carrington *et al.*, 2016 ▸); (*i*) 2-(2′-pyrid­yl)benzimidazole (Tzeng *et al.*, 2011 ▸); (*j*) acetyl­pyridine benzoyl­hydrazone (Grewe *et al.*, 2003 ▸).

**Table 3:** Experimental details

Crystal data	
Chemical formula	[Re(C_19_H_13_N_3_S)(CO)_3_(H_2_O)]PF_6_·CH_4_O
*M*_r_	780.64
Crystal system, space group	Triclinic, *P* 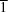
Temperature (K)	160
*a*, *b*, *c* (Å)	10.0447 (3), 10.7580 (3), 13.6263 (4)
α, β, γ (°)	74.335 (1), 76.285 (1), 68.874 (1)
*V* (Å^3^)	1306.38 (7)
*Z*	2
Radiation type	Mo *K*α
μ (mm^−1^)	4.88
Crystal size (mm)	0.48 × 0.26 × 0.04

Data collection	
Diffractometer	Rigaku R-AXIS SPIDER IPDS
Absorption correction	Numerical (*CrystalClear*; Rigaku, 2005 ▸)
*T*_min_, *T*_max_	0.496, 1.000
No. of measured, independent and observed [*I* > 2σ(*I*)] reflections	25647, 5694, 5416
*R*_int_	0.027
(sin θ/λ)_max_ (Å^−1^)	0.639

Refinement	
*R*[*F* ^2^ > 2σ(*F* ^2^)], *wR*(*F* ^2^), *S*	0.020, 0.045, 1.06
No. of reflections	5694
No. of parameters	437
H-atom treatment	All H-atom parameters refined
Δρ_max_, Δρ_min_ (e Å^−3^)	0.97, −0.53

Computer programs: *CrystalClear* (Rigaku, 2005 ▸), *SHELXS* (Sheldrick, 2015*a* ▸), *SHELXL2014/6* (Sheldrick, 2015*b* ▸), *DIAMOND* (Crystal Impact, 2012 ▸) and *publCIF* (Westrip, 2010 ▸).

## supplementary crystallographic information

### Crystal data

**Table:**

[Re(C_19_H_13_N_3_S)(CO)_3_(H_2_O)]PF_6_·CH_4_O	*Z* = 2
*M**_r_* = 780.64	*F*(000) = 756
Triclinic, *P*1	*D*_x_ = 1.985 Mg m^−^^3^
*a* = 10.0447 (3) Å	Mo *K*α radiation, λ = 0.71073 Å
*b* = 10.7580 (3) Å	Cell parameters from 23889 reflections
*c* = 13.6263 (4) Å	θ = 3.2–27.5°
α = 74.335 (1)°	µ = 4.88 mm^−^^1^
β = 76.285 (1)°	*T* = 160 K
γ = 68.874 (1)°	Parallelepiped, red brown
*V* = 1306.38 (7) Å^3^	0.48 × 0.26 × 0.04 mm

### Data collection

**Table:**

Rigaku R-AXIS SPIDER IPDS diffractometer	5416 reflections with *I* > 2σ(*I*)
Radiation source: fine-focus sealed tube	*R*_int_ = 0.027
θ scans	θ_max_ = 27.0°, θ_min_ = 3.1°
Absorption correction: numerical (*CrystalClear*; Rigaku, 2005)	*h* = −12→12
*T*_min_ = 0.496, *T*_max_ = 1.000	*k* = −13→13
25647 measured reflections	*l* = −17→16
5694 independent reflections	

### Refinement

**Table:**

Refinement on *F*^2^	0 restraints
Least-squares matrix: full	Hydrogen site location: difference Fourier map
*R*[*F*^2^ > 2σ(*F*^2^)] = 0.020	All H-atom parameters refined
*wR*(*F*^2^) = 0.045	*w* = 1/[σ^2^(*F*_o_^2^) + (0.0212*P*)^2^ + 1.3806*P*] where *P* = (*F*_o_^2^ + 2*F*_c_^2^)/3
*S* = 1.06	(Δ/σ)_max_ = 0.002
5694 reflections	Δρ_max_ = 0.97 e Å^−^^3^
437 parameters	Δρ_min_ = −0.53 e Å^−^^3^

### Special details

**Table:**

Geometry. All esds (except the esd in the dihedral angle between two l.s. planes) are estimated using the full covariance matrix. The cell esds are taken into account individually in the estimation of esds in distances, angles and torsion angles; correlations between esds in cell parameters are only used when they are defined by crystal symmetry. An approximate (isotropic) treatment of cell esds is used for estimating esds involving l.s. planes.

### Fractional atomic coordinates and isotropic or equivalent isotropic displacement parameters (Å^2^)

**Table:**

	*x*	*y*	*z*	*U*_iso_*/*U*_eq_	
Re1	0.60782 (2)	0.80579 (2)	0.72803 (2)	0.02076 (4)	
O1W	0.7342 (2)	0.6297 (2)	0.82936 (19)	0.0305 (4)	
C1	0.7598 (3)	0.7903 (3)	0.6110 (2)	0.0278 (6)	
O1	0.8494 (2)	0.7844 (2)	0.54080 (17)	0.0378 (5)	
C2	0.6724 (3)	0.9386 (3)	0.7560 (2)	0.0274 (6)	
O2	0.7081 (2)	1.0222 (2)	0.76923 (17)	0.0368 (5)	
C3	0.4873 (3)	0.9562 (3)	0.6452 (2)	0.0270 (6)	
O3	0.4169 (2)	1.0526 (2)	0.59636 (17)	0.0384 (5)	
N1	0.4410 (2)	0.7968 (2)	0.86255 (17)	0.0235 (5)	
N2	0.5265 (2)	0.6456 (2)	0.71983 (17)	0.0225 (4)	
N3	0.8451 (2)	0.3276 (2)	0.34299 (18)	0.0255 (5)	
S1	0.76531 (10)	0.12865 (8)	0.46521 (7)	0.0419 (2)	
C4	0.3971 (3)	0.8740 (3)	0.9337 (2)	0.0286 (6)	
C5	0.3005 (3)	0.8508 (3)	1.0224 (2)	0.0327 (6)	
C6	0.2477 (4)	0.7445 (3)	1.0389 (2)	0.0362 (7)	
C7	0.2894 (3)	0.6653 (3)	0.9652 (2)	0.0323 (6)	
C8	0.3849 (3)	0.6940 (3)	0.8781 (2)	0.0256 (5)	
C9	0.4331 (3)	0.6167 (3)	0.7970 (2)	0.0258 (6)	
C10	0.5866 (3)	0.5565 (3)	0.6476 (2)	0.0230 (5)	
C11	0.6169 (3)	0.4165 (3)	0.6826 (2)	0.0265 (6)	
C12	0.6798 (3)	0.3310 (3)	0.6132 (2)	0.0286 (6)	
C13	0.7123 (3)	0.3835 (3)	0.5087 (2)	0.0244 (5)	
C14	0.6791 (3)	0.5242 (3)	0.4738 (2)	0.0249 (5)	
C15	0.6175 (3)	0.6105 (3)	0.5431 (2)	0.0233 (5)	
C16	0.7791 (3)	0.2926 (3)	0.4342 (2)	0.0257 (6)	
C17	0.8562 (3)	0.1046 (3)	0.3433 (2)	0.0330 (7)	
C18	0.8893 (4)	−0.0061 (3)	0.2976 (3)	0.0441 (8)	
C19	0.9563 (4)	0.0040 (3)	0.1968 (3)	0.0415 (8)	
C20	0.9923 (3)	0.1196 (3)	0.1420 (3)	0.0368 (7)	
C21	0.9609 (3)	0.2297 (3)	0.1873 (2)	0.0328 (6)	
C22	0.8905 (3)	0.2234 (3)	0.2887 (2)	0.0272 (6)	
C1M	0.9995 (5)	0.3997 (5)	0.6743 (4)	0.0528 (10)	
O1M	0.9675 (3)	0.5110 (2)	0.7203 (2)	0.0505 (7)	
P1	0.72699 (9)	0.71646 (8)	1.09118 (6)	0.03265 (17)	
F1	0.8471 (3)	0.6216 (3)	1.0214 (3)	0.0957 (10)	
F2	0.8364 (4)	0.7745 (3)	1.1108 (2)	0.0963 (11)	
F3	0.7504 (3)	0.5986 (2)	1.19014 (18)	0.0677 (7)	
F4	0.6144 (3)	0.6560 (2)	1.0673 (2)	0.0683 (7)	
F5	0.6990 (3)	0.8341 (2)	0.99029 (18)	0.0631 (6)	
F6	0.5955 (4)	0.8096 (3)	1.1538 (3)	0.1039 (12)	
H4	0.432 (3)	0.942 (3)	0.920 (3)	0.033 (9)*	
H5	0.276 (4)	0.906 (4)	1.069 (3)	0.042 (10)*	
H6	0.186 (4)	0.731 (3)	1.094 (3)	0.031 (8)*	
H7	0.257 (4)	0.596 (4)	0.970 (3)	0.044 (10)*	
H9	0.398 (3)	0.546 (3)	0.801 (2)	0.028 (8)*	
H11	0.598 (3)	0.384 (3)	0.750 (2)	0.023 (7)*	
H12	0.704 (3)	0.237 (3)	0.634 (3)	0.034 (8)*	
H14	0.696 (3)	0.560 (3)	0.406 (3)	0.026 (8)*	
H15	0.592 (3)	0.702 (3)	0.517 (2)	0.016 (7)*	
H18	0.868 (4)	−0.083 (4)	0.334 (3)	0.043 (10)*	
H19	0.976 (4)	−0.070 (4)	0.170 (3)	0.045 (10)*	
H20	1.038 (4)	0.127 (3)	0.071 (3)	0.036 (9)*	
H21	0.984 (3)	0.311 (3)	0.149 (3)	0.031 (8)*	
H101	0.823 (5)	0.581 (4)	0.801 (3)	0.051 (11)*	
H102	0.749 (4)	0.641 (4)	0.874 (3)	0.044 (12)*	
H201	1.041 (5)	0.541 (5)	0.709 (4)	0.073 (14)*	
H202	1.091 (6)	0.323 (5)	0.698 (4)	0.099 (18)*	
H203	0.915 (6)	0.364 (5)	0.702 (4)	0.095 (17)*	
H204	1.008 (6)	0.427 (6)	0.607 (5)	0.10 (2)*	

### Atomic displacement parameters (Å^2^)

**Table:**

	*U*^11^	*U*^22^	*U*^33^	*U*^12^	*U*^13^	*U*^23^
Re1	0.02758 (6)	0.01809 (5)	0.01851 (6)	−0.01022 (4)	−0.00178 (4)	−0.00422 (4)
O1W	0.0366 (12)	0.0287 (10)	0.0275 (12)	−0.0099 (9)	−0.0071 (10)	−0.0065 (9)
C1	0.0326 (14)	0.0231 (13)	0.0306 (15)	−0.0109 (11)	−0.0065 (13)	−0.0058 (11)
O1	0.0379 (12)	0.0408 (12)	0.0309 (12)	−0.0142 (10)	0.0052 (10)	−0.0079 (10)
C2	0.0333 (14)	0.0257 (13)	0.0225 (14)	−0.0106 (11)	−0.0041 (11)	−0.0020 (11)
O2	0.0531 (13)	0.0295 (10)	0.0382 (12)	−0.0228 (10)	−0.0122 (10)	−0.0056 (9)
C3	0.0338 (14)	0.0258 (13)	0.0227 (14)	−0.0134 (12)	0.0003 (11)	−0.0057 (11)
O3	0.0427 (12)	0.0308 (11)	0.0359 (12)	−0.0098 (10)	−0.0094 (10)	0.0028 (10)
N1	0.0271 (11)	0.0211 (10)	0.0220 (11)	−0.0069 (9)	−0.0025 (9)	−0.0059 (9)
N2	0.0285 (11)	0.0186 (10)	0.0227 (11)	−0.0089 (9)	−0.0038 (9)	−0.0062 (9)
N3	0.0274 (11)	0.0236 (11)	0.0270 (12)	−0.0093 (9)	−0.0015 (9)	−0.0084 (9)
S1	0.0606 (5)	0.0262 (3)	0.0392 (4)	−0.0243 (4)	0.0199 (4)	−0.0164 (3)
C4	0.0339 (15)	0.0248 (13)	0.0285 (15)	−0.0084 (12)	−0.0040 (12)	−0.0101 (11)
C5	0.0376 (16)	0.0338 (15)	0.0259 (15)	−0.0066 (13)	−0.0031 (12)	−0.0130 (13)
C6	0.0394 (17)	0.0394 (16)	0.0242 (15)	−0.0125 (14)	0.0069 (13)	−0.0080 (13)
C7	0.0364 (16)	0.0297 (14)	0.0309 (16)	−0.0155 (13)	0.0034 (13)	−0.0069 (12)
C8	0.0309 (14)	0.0218 (12)	0.0245 (14)	−0.0101 (11)	−0.0021 (11)	−0.0049 (11)
C9	0.0326 (14)	0.0231 (13)	0.0251 (14)	−0.0148 (11)	0.0003 (11)	−0.0061 (11)
C10	0.0261 (13)	0.0222 (12)	0.0238 (13)	−0.0106 (10)	−0.0014 (10)	−0.0078 (10)
C11	0.0385 (15)	0.0224 (13)	0.0186 (13)	−0.0126 (11)	−0.0013 (11)	−0.0026 (11)
C12	0.0397 (15)	0.0183 (12)	0.0286 (15)	−0.0118 (11)	−0.0028 (12)	−0.0045 (11)
C13	0.0269 (13)	0.0241 (12)	0.0251 (14)	−0.0120 (10)	0.0008 (11)	−0.0084 (11)
C14	0.0308 (14)	0.0239 (13)	0.0217 (14)	−0.0118 (11)	−0.0019 (11)	−0.0053 (11)
C15	0.0293 (13)	0.0186 (12)	0.0233 (13)	−0.0099 (10)	−0.0037 (11)	−0.0035 (10)
C16	0.0294 (13)	0.0202 (12)	0.0290 (14)	−0.0099 (10)	−0.0018 (11)	−0.0072 (11)
C17	0.0374 (15)	0.0282 (14)	0.0351 (16)	−0.0153 (12)	0.0090 (13)	−0.0152 (12)
C18	0.0501 (19)	0.0326 (16)	0.052 (2)	−0.0214 (15)	0.0175 (16)	−0.0228 (15)
C19	0.0375 (17)	0.0401 (17)	0.052 (2)	−0.0133 (14)	0.0089 (15)	−0.0310 (16)
C20	0.0329 (15)	0.0462 (18)	0.0336 (17)	−0.0127 (14)	0.0054 (13)	−0.0205 (14)
C21	0.0349 (15)	0.0338 (15)	0.0311 (16)	−0.0139 (13)	0.0009 (13)	−0.0098 (13)
C22	0.0255 (13)	0.0274 (13)	0.0316 (15)	−0.0104 (11)	−0.0004 (11)	−0.0112 (12)
C1M	0.049 (2)	0.058 (2)	0.066 (3)	−0.0270 (19)	−0.001 (2)	−0.029 (2)
O1M	0.0334 (12)	0.0402 (13)	0.081 (2)	−0.0137 (10)	0.0051 (12)	−0.0265 (13)
P1	0.0454 (4)	0.0270 (4)	0.0253 (4)	−0.0160 (3)	−0.0040 (3)	−0.0003 (3)
F1	0.088 (2)	0.0651 (16)	0.104 (2)	−0.0127 (15)	0.0385 (17)	−0.0299 (16)
F2	0.147 (3)	0.108 (2)	0.0777 (19)	−0.102 (2)	−0.0657 (19)	0.0366 (17)
F3	0.0950 (18)	0.0641 (14)	0.0560 (14)	−0.0505 (14)	−0.0404 (13)	0.0284 (12)
F4	0.0874 (17)	0.0592 (14)	0.0723 (16)	−0.0459 (13)	−0.0405 (14)	0.0199 (12)
F5	0.0817 (16)	0.0568 (13)	0.0504 (13)	−0.0385 (12)	−0.0226 (12)	0.0233 (11)
F6	0.130 (3)	0.0520 (15)	0.096 (2)	−0.0228 (16)	0.052 (2)	−0.0301 (15)

### Geometric parameters (Å, º)

**Table:**

Re1—C3	1.899 (3)	C11—C12	1.380 (4)
Re1—C2	1.920 (3)	C11—H11	0.89 (3)
Re1—C1	1.925 (3)	C12—C13	1.389 (4)
Re1—N1	2.177 (2)	C12—H12	0.93 (3)
Re1—O1W	2.189 (2)	C13—C14	1.397 (4)
Re1—N2	2.194 (2)	C13—C16	1.475 (4)
O1W—H101	0.91 (4)	C14—C15	1.386 (4)
O1W—H102	0.72 (4)	C14—H14	0.90 (3)
C1—O1	1.146 (4)	C15—H15	0.92 (3)
C2—O2	1.150 (3)	C17—C18	1.390 (4)
C3—O3	1.158 (3)	C17—C22	1.410 (4)
N1—C4	1.339 (3)	C18—C19	1.373 (5)
N1—C8	1.361 (3)	C18—H18	0.92 (4)
N2—C9	1.284 (3)	C19—C20	1.389 (5)
N2—C10	1.436 (3)	C19—H19	0.90 (4)
N3—C16	1.289 (4)	C20—C21	1.384 (4)
N3—C22	1.390 (3)	C20—H20	0.96 (3)
S1—C17	1.733 (3)	C21—C22	1.390 (4)
S1—C16	1.748 (3)	C21—H21	0.96 (3)
C4—C5	1.385 (4)	C1M—O1M	1.401 (4)
C4—H4	0.89 (3)	C1M—H202	1.04 (6)
C5—C6	1.372 (4)	C1M—H203	1.01 (6)
C5—H5	0.91 (4)	C1M—H204	0.87 (6)
C6—C7	1.385 (4)	O1M—H201	0.88 (5)
C6—H6	0.87 (3)	P1—F2	1.547 (2)
C7—C8	1.378 (4)	P1—F6	1.565 (3)
C7—H7	0.89 (4)	P1—F1	1.579 (3)
C8—C9	1.450 (4)	P1—F3	1.579 (2)
C9—H9	0.94 (3)	P1—F5	1.600 (2)
C10—C15	1.392 (4)	P1—F4	1.621 (2)
C10—C11	1.393 (4)		
			
C3—Re1—C2	85.26 (12)	C11—C12—H12	122 (2)
C3—Re1—C1	89.26 (12)	C13—C12—H12	118 (2)
C2—Re1—C1	87.63 (12)	C12—C13—C14	119.5 (2)
C3—Re1—N1	95.27 (10)	C12—C13—C16	120.8 (2)
C2—Re1—N1	98.04 (10)	C14—C13—C16	119.7 (2)
C1—Re1—N1	173.00 (9)	C15—C14—C13	120.2 (3)
C3—Re1—O1W	176.33 (10)	C15—C14—H14	119.4 (19)
C2—Re1—O1W	96.59 (10)	C13—C14—H14	120.3 (19)
C1—Re1—O1W	93.97 (10)	C14—C15—C10	119.7 (2)
N1—Re1—O1W	81.35 (8)	C14—C15—H15	118.1 (18)
C3—Re1—N2	99.29 (10)	C10—C15—H15	122.0 (18)
C2—Re1—N2	171.90 (10)	N3—C16—C13	124.0 (2)
C1—Re1—N2	99.07 (10)	N3—C16—S1	115.7 (2)
N1—Re1—N2	74.96 (8)	C13—C16—S1	120.2 (2)
O1W—Re1—N2	78.50 (8)	C18—C17—C22	121.5 (3)
Re1—O1W—H101	118 (2)	C18—C17—S1	129.6 (2)
Re1—O1W—H102	117 (3)	C22—C17—S1	108.8 (2)
H101—O1W—H102	102 (4)	C19—C18—C17	117.7 (3)
O1—C1—Re1	178.4 (2)	C19—C18—H18	122 (2)
O2—C2—Re1	177.0 (2)	C17—C18—H18	121 (2)
O3—C3—Re1	176.2 (2)	C18—C19—C20	121.6 (3)
C4—N1—C8	117.9 (2)	C18—C19—H19	115 (2)
C4—N1—Re1	127.09 (19)	C20—C19—H19	123 (2)
C8—N1—Re1	114.86 (17)	C21—C20—C19	120.9 (3)
C9—N2—C10	118.0 (2)	C21—C20—H20	117 (2)
C9—N2—Re1	115.25 (18)	C19—C20—H20	122 (2)
C10—N2—Re1	125.69 (16)	C20—C21—C22	118.7 (3)
C16—N3—C22	111.1 (2)	C20—C21—H21	121.0 (19)
C17—S1—C16	89.41 (13)	C22—C21—H21	120.2 (19)
N1—C4—C5	122.5 (3)	N3—C22—C21	125.6 (3)
N1—C4—H4	116 (2)	N3—C22—C17	114.9 (2)
C5—C4—H4	122 (2)	C21—C22—C17	119.4 (3)
C6—C5—C4	119.2 (3)	O1M—C1M—H202	111 (3)
C6—C5—H5	122 (2)	O1M—C1M—H203	105 (3)
C4—C5—H5	119 (2)	H202—C1M—H203	108 (4)
C5—C6—C7	119.1 (3)	O1M—C1M—H204	109 (4)
C5—C6—H6	119 (2)	H202—C1M—H204	113 (5)
C7—C6—H6	122 (2)	H203—C1M—H204	111 (5)
C8—C7—C6	119.0 (3)	C1M—O1M—H201	112 (3)
C8—C7—H7	117 (2)	F2—P1—F6	93.4 (2)
C6—C7—H7	124 (2)	F2—P1—F1	92.4 (2)
N1—C8—C7	122.2 (3)	F6—P1—F1	173.6 (2)
N1—C8—C9	115.3 (2)	F2—P1—F3	92.81 (13)
C7—C8—C9	122.4 (2)	F6—P1—F3	91.03 (17)
N2—C9—C8	119.2 (2)	F1—P1—F3	91.27 (16)
N2—C9—H9	120.4 (19)	F2—P1—F5	89.19 (13)
C8—C9—H9	120.4 (19)	F6—P1—F5	88.71 (16)
C15—C10—C11	120.3 (2)	F1—P1—F5	88.78 (16)
C15—C10—N2	119.7 (2)	F3—P1—F5	177.99 (13)
C11—C10—N2	120.0 (2)	F2—P1—F4	178.45 (17)
C12—C11—C10	119.7 (3)	F6—P1—F4	87.72 (18)
C12—C11—H11	121.4 (19)	F1—P1—F4	86.42 (17)
C10—C11—H11	118.8 (19)	F3—P1—F4	88.27 (12)
C11—C12—C13	120.6 (2)	F5—P1—F4	89.73 (12)

### Hydrogen-bond geometry (Å, º)

**Table:**

*D*—H···*A*	*D*—H	H···*A*	*D*···*A*	*D*—H···*A*
C5—H5···O2^i^	0.91 (4)	2.59 (4)	3.439 (4)	156 (3)
C9—H9···F3^ii^	0.94 (3)	2.47 (3)	3.390 (3)	166 (2)
O1*W*—H101···O1*M*	0.91 (4)	1.67 (4)	2.558 (3)	165 (4)
O1*W*—H102···F1	0.72 (4)	2.36 (4)	3.059 (5)	164 (4)
O1*M*—H201···N3^iii^	0.88 (5)	2.01 (5)	2.842 (3)	158 (4)

Symmetry codes: (i) −*x*+1, −*y*+2, −*z*+2; (ii) −*x*+1, −*y*+1, −*z*+2; (iii) −*x*+2, −*y*+1, −*z*+1.
